# LipidClock: A Lipid-Based Predictor of Biological Age

**DOI:** 10.3389/fragi.2022.828239

**Published:** 2022-05-27

**Authors:** Maximilian Unfried, Li Fang Ng, Amaury Cazenave-Gassiot, Krishna Chaithanya Batchu, Brian K. Kennedy, Markus R. Wenk, Nicholas Tolwinski, Jan Gruber

**Affiliations:** ^1^ Department of Biochemistry, Yong Loo Lin School of Medicine, National University of Singapore, Singapore, Singapore; ^2^ Healthy Longevity Translational Research Program, Yong Loo Lin School of Medicine, National University of Singapore, Singapore, Singapore; ^3^ Science Divisions, Yale-NUS College, Singapore, Singapore; ^4^ Singapore Lipidomics Incubator, Life Sciences Institute, National University of Singapore, Singapore, Singapore

**Keywords:** aging, lipidomics, lipids, machine learning, biomarker, aging clock, *Caenorhabditis elegans*

## Abstract

Complexity is a fundamental feature of biological systems. Omics techniques like lipidomics can simultaneously quantify many thousands of molecules, thereby directly capturing the underlying biological complexity. However, this approach transfers the original biological complexity to the resulting datasets, posing challenges in data reduction and analysis. Aging is a prime example of a process that exhibits complex behaviour across multiple scales of biological organisation. The aging process is characterised by slow, cumulative and detrimental changes that are driven by intrinsic biological stochasticity and mediated through non-linear interactions and feedback within and between these levels of organization (ranging from metabolites, macromolecules, organelles and cells to tissue and organs). Only collectively and over long timeframes do these changes manifest as the exponential increases in morbidity and mortality that define biological aging, making aging a problem more difficult to study than the aetiologies of specific diseases. But aging’s time dependence can also be exploited to extract key insights into its underlying biology. Here we explore this idea by using data on changes in lipid composition across the lifespan of an organism to construct and test a LipidClock to predict biological age in the nematode *Caenorhabdits elegans*. The LipidClock consist of a feature transformation *via* Principal Component Analysis followed by Elastic Net regression and yields and Mean Absolute Error of 1.45 days for wild type animals and 4.13 days when applied to mutant strains with lifespans that are substantially different from that of wild type. Gompertz aging rates predicted by the LipidClock can be used to simulate survival curves that are in agreement with those from lifespan experiments.

## Introduction

Biological aging is defined by an exponential increase in mortality and a concomitant, dramatic increase in the risk of age-dependent diseases, including cancer, cardiovascular and neurodegenerative diseases. However, it is becoming increasingly clear that individual biological aging rates are not fixed and that subjects having identical chronological ages can have very different biological ages (Jylhävä et al., 2017). Biological aging is impacted by genetics, lifestyle factors, diet, intrinsic stochasticity, and drug interventions (Kennedy et al., 2014). Tools to objectively quantify differences in biological aging rate and of biological age are therefore critical, not only to further our understanding of biological aging itself but also to the identification and testing of age-modifying medical and lifestyle interventions. Such tools are referred to as “Aging Clocks” in the field of aging and geroscience. Ageing clocks infer biological age, which is typically not identical with chronological age. The ultimate goal are aging “clocks,” that are able to predict future morbidity and all-cause mortality on an individual level and with high precision.

The most prominent type of aging clock was originally described by Horvath in 2013 (Horvath 2013) based on changes in DNA methylation (DNAm). Since then, methylation clocks have seen extensive development for use in diverse human and animal tissues and have gone through several iterations of refinement and validation (Horvath and Raj, 2018; Bell et al., 2019; Noroozi et al., 2021). There is now a repertoire of clocks to choose from and clocks are widely deployed, including by several commercial ventures (Horvath and Raj, 2018). Second generation methylation clocks have reached a high level of precision and accuracy and have been validated for their predictive power towards morbidity and mortality, with clocks such a PhenoAge (Levine et al., 2018) and GrimAge (Lu et al., 2019).

Methylation clocks are regarded as the gold-standard for quantitative determination of biological age. However, connecting DNA methylation to the underlying biological mechanisms of aging remains challenging and this is an active area of research (Levine, 2019).

However, in principle, aging clocks can be constructed using many types of high dimensional data, as long as such data maps to the physiological aging “state” of an organism. Indeed, aging clocks have been created based on panels of clinical markers, including routine clinical chemistry (Liu et al., 2018), transcriptomic signatures (Mamoshina et al., 2018; Meyer and Schumacher, 2021), metabolomic profiles (Robinson et al., 2020; Hwangbo et al., 2021), inflammation (Sayed et al., 2021), facial images (Bobrov et al., 2018; Xia et al., 2020), activity data from commercial fitness trackers (Pyrkov et al., 2018) and changes in gut microbiome (Galkin et al., 2020).

Clocks based on different types of data have distinct advantages and disadvantages. Clocks based directly on readouts of physiological and molecular state, such as transcriptomic (Mamoshina et al., 2018) and metabolomic clocks (Robinson et al., 2020; Hwangbo et al., 2021) can be more readily interpreted when the aim is to extract actionable insights, such as specific pathways or biological processes to target with interventions. For example, transcriptomic clocks such as BiT age analyze gene expression profiles with the aim of identifying predictive gene sets. Identification of gene sets is then followed by functional enrichment analysis to extract specific biological processes involved in aging with potential value as targets in age modulation (Meyer and Schumacher, 2021).

Similarly, Robinson *et al.* have developed a clock using untargeted metabolomics, which was able to detect metabolomic age acceleration in individuals as a function of obesity, diabetes, heavy alcohol use or depression (Robinson et al., 2020). Using metabolic pathway enrichment, the authors were then able to identify contributing mechanisms, including altered intracellular communication involving tryptophan, tyrosine and biopterin metabolic pathways and mitochondrial dysfunction.

An attractive class of molecules to investigate in this context are biological lipids. Lipids are centrally involved in many physiological processes. They play pivotal roles in signaling (Fernandis and Wenk, 2007), membrane stability and fluidity (Seu et al., 2006), as well as energy storage (Welte and Gould, 2017). Lipids are a diverse class of molecules, comprising species from 8 different broad lipid families (Sud et al., 2007). In human blood serum alone, over 3,000 lipids have been identified and quantified (Psychogios et al., 2011; Quehenberger and Dennis, 2011). Furthermore, specific blood lipids and fatty acids are already well-established and routinely used clinical markers for cardiovascular and metabolic diseases (Arsenault et al., 2011). A dysregulated lipid metabolism is connected to many disease states including neurodegenerative diseases (Lim et al., 2020), cardiovascular diseases (Ridker et al., 2005) and cancer (Shah et al., 2008).

Modulation of lipid metabolism can also directly impact aging. For instance, work in our lab has demonstrated that lifespan and healthspan benefits of interventions based on synergistic interactions between certain drugs require SREBP-dependent changes in lipid metabolism, including changes in mono-unsaturated fatty acid (MUFA) and medium-chain-fatty-acyl-containing (TAGs) content (Admasu et al., 2018).

The lipidome is highly dynamic and known to change significantly with age (Gonzalez-Covarrubias et al., 2013), health status (de Diego et al., 2019) and diet (Boretti et al., 2019). Several lipid classes and sub-categories appear to undergo systematic changes with age (de Diego et al., 2019). A 2020 longitudinal multiomics study found that 40% of the metabolites that correlated with age where lipids (Ahadi et al., 2020). In human plasma, ceramides have been reported to increase with age (Mielke et al., 2015) while blood lysophosphatidylcholine levels show a decline (Mapstone et al., 2014). A lipid class that appears to stay constant and does not show age-related changes is the endocannabinoid anandamide which is involved in signaling in the brain (Piyanova et al., 2015). Certain lipid signatures have been linked to longevity in humans and animals. For instance, in the blood plasma of centenarians, higher levels of sphingolipids have been observed (Montoliu et al., 2014). A signature of 19 lipid features, including increased levels of phosphocholine and sphingomyelin and a decrease in phosphatidylethanolamine and long-chain triglycerides, has been associated with familial longevity in women (Gonzalez-Covarrubias et al., 2013). In the animal kingdom, it appears that odd chain fatty acids and ether lipids are increased in long-lived animals relative to shorter-lived ones (de Diego et al., 2019).

The evidence for abundant age-dependent changes in molecular lipid composition and their established relationship to health and disease suggest that lipids might represent an exciting target for the development of aging clocks, similar to other “-omics” aging clocks. Here we report proof-of-principle development and validation of one such a lipidomic clock in the model organism *Caenorhabditis elegans*. We demonstrate that our clock can predict the aging rate of WT and mutant *C. elegans* based on their lipid profile alone.

## Materials and Methods

### *C. elegans* Sample Preparation

The data used to develop the LipidClock were derived from cohorts of aging *C. elegans*, sampled at different time points. To validate the lipid clock, we compared animals of four different strains that are characterized by different lifespans. We used standard wild-type (WT) Bristol N2 as controls undergoing normal aging. We utilized *mev-1* as short-lived strain. *Mev-1* animals carry a defect in complex II of the electron transfer chain (ETC), produce excessive amounts of reactive oxygen species, and suffer from elevated oxidative damage, as well as defective energy metabolism have shorter lifespans (Ishii et al., 1998; Senoo-Matsuda et al., 2001). We selected two strains with extended lifespan and healthspans; *age-1* and *eat-2*. Lifespan is extended by different mechanisms in these two strains. *Age-1* carries a nonsense mutation in phosphatidylinositol 3-kinase (PI3K), a member of the insulin-like growth-factor axis (Friedman and Johnson, 1988). *Eat-2* animals have reduced pharyngeal pumping rates, resulting in decreased food intake and extended lifespan by a mechanism related to caloric restriction (Lakowski and Hekimi, 1998). We obtained hermaphrodite *C. elegans* strains of wild-type N2, DA1116: *eat-2* (*ad1116*), TJ1062: *spe-9* (*hc88*) *I*; *rrf-3* (*b26*) *age-1* (*hx542*), and TK22: *mev-1* (*kn1*) *III* from the *Caenorhabditis* Genetic Center. Cohorts were synchronized using the standard alkaline hypochlorite approach (Stiernagle, 2006). For WT and mutant strains, nematodes were grown and maintained on standard Nematode Growth Medium (NGM) agar plates at 20 °C with *E. coli* OP50-1 bacteria as a food source as previously described (Admasu et al., 2018). Age-synchronized adult worms were transferred to a fresh NGM plate containing 5-fluoro-2′- deoxyuridine (FUdR) to prevent egg hatching and overcrowding by progeny. Samples from aging cohorts were collected for each strain at 3-, 5-, 10-, 15-, and 20-days post hatching. However, for the short-lived *mev-1* strain we found that a significant number of animals start to die after day 10 of age and we therefore only collected samples up to day 10 for this strain. On each sampling day, between 2000 and 3,000 worms were collected from each cohort by washing culture plates with M9 buffer. Three separate samples were collected on for each strain and age.

### Two-Phase Lipid Extraction for MS Analysis

Samples were processed as previously described (Admasu et al., 2018). Briefly, worm pellets were transferred to 2 ml polypropylene tubes containing 250 µL lysis buffer (20 mM Tris- HCl pH 7.4, 100 mM NaCl, 0.5 mM EDTA, 5% glycerol). Metallic beads were added to each tube before incubation on ice for 15 min followed by homogenization using a bead beater (Precellys, France). Homogenates were extracted using a Folch extraction (Folch et al., 1957). For quantification purposes, extraction solvents were spiked with internal standards PC 34:0, PE 28:0, lysoPC 20:0, lysoPE 14:0, PS 34:0, PG 34:0, SM 30:1, and Cer 35:1 (Avanti Polar Lipids). To each tube, 0.5% of butylated hydroxytoluene (BHT) was added to prevent lipid oxidation during extraction. After phase separation, the organic phase was transferred to a fresh centrifuge tube and dried under vacuum using a vacuum concentrator (SpeedVac, Thermo Savant, Milford, United States). Finally, dried lipid extracts were reconstituted in 100 µl methanol and kept at −80°C until the MS analysis.

### Data Acquisition

Lipidomics analysis was performed as previously described (Admasu et al., 2018). Briefly, we used an Agilent 1260-Ultra Performance Liquid chromatography (UPLC) system coupled to Triple Quad Mass spectrometer (Agilent 6,490) with dynamic multiple reaction monitoring (dMRM) for lipid quantification. The UPLC system was equipped with a Waters ACQUITY BEH C18 column (1.0 × 100 mm). Solvent A was acetonitrile/H_2_O (60:40) with 10 mM ammonium formate and 1% formic acid. Solvent B was isopropanol/acetonitrile (90:10) containing 10 mM ammonium formate and 1% formic acid. Gradient elution was performed initially from 40 to 100% solvent B over 14 min at a flow rate was 0.13 ml/min and a column temperature of 60°C. After 3 min at 100%, solvent B was decreased rapidly back to 40% in 1 min and this was then maintained until the end of the run at 20 min. The eluent was directed to the ESI source of the mass spectrometer operated in the positive ion mode. The MS conditions were as follows: For ESI: gas temperature, 300 °C; gas flow, 10 L/min; sheath gas temperature, 350 °C; sheath gas flow, 8 l/min; and capillary voltage, 3,500 V.

### MS Data Analysis

Data processing, including peak smoothing and integration of area under the curve for each transition measured, was performed using the MassHunter Quantitative B.08.00 (Agilent). Manual inspection of raw peaks was carried out to ensure correct peak picking, and the peak area data were exported in csv format for further analysis. The peak area of each endogenous lipid species was normalized to the corresponding class-specific IS for quantitation. To ensure the accuracy and reproducibility, pooled quality controls samples were analysed throughout the analytical sequence (once in between every 10 experimental samples) and coefficients of variation (CoV) were calculated for all 238 dMRM transitions. Only those MRM transitions that had CoV <25% were used in subsequent analysis. Overall, 168 lipid species were reliably quantified. Of these, 26 were ceramides (Cer), 23 lysophosphatidylcholines (LPC), 11 ether-linked lysophosphatidylcholines (LPC-O), 10 lysophosphatidylethanolamine (LPE), 41 phosphatidylcholine (PC), 19 phosphatidylethanolamine (PE), 5 phosphatidylglycerol (PG), 13 phosphatidylserine (PS), and 20 Sphingomyelin (SM). The total dataset comprised data on 168 lipid species, for 54 samples covering three mutant strains and wild type at ages between 3 and 20 days (Table S2). The data will be available on GitHub (https://github.com/max-unfried/lipid-clock).

## Statistical Methods

### Principal Component Analysis

At the core of the LipidClock is a coordinate transformation by Principal Component Analysis (PCA) followed by supervised learning *via* Elastic net regression**,** an approach similar to the rat epigenetic clock by Levine et al. (2020), in which PC1 strongly correlates with age**.** PCA is a method widely used for linear dimensionality reduction and feature extraction. It is a Eigenvalue method that is used to factorize a matrix into its principal components (PCs), which can be used as features instead of the original data to train machine learning models. PCA rotates the coordinate system of feature space (lipid species) to align the main axes with directions in feature space along which the covariance between samples is maximal. Here, we first normalized lipid data by dividing the abundance of each lipid species by the median for young control animals collected during the same run. To give equal weight to increases and decreases in lipid abundance, we then converted lipid abundances into log-2 fold changes vs the mean for each lipid species across all experiments. We then carried out PCA on this log-2 fold change data matrix, resulting in a new base for the feature (log-fold change in lipids) space. The original data comprised 168 lipid species, observed across 54 samples. The PCA therefore yields 54 singular vectors or principal components (PCs) (Strang, 2016). The singular vectors are an orthonormal base of the subspace of the full feature space in which the 54 samples are contained. Lipid changes for each sample (experimental condition) can therefore be expressed in terms of the 54 PCs. We transform each sample into these PC coordinates before training the aging clock regression model.

### Elastic Net Regression

Standard linear regression can yield individual estimated coefficients that are large and this can destabilize the model. A way to counteract this is to penalize the model with a sum of squared coefficient values. This penalty is called L2-penalty and has the effect of minimizing the size of all coefficients. Another way of penalizing the model is based on the absolute sum of coefficients (L1-penalty). The L1-penalty minimizes the size of all coefficients and allows for coefficients to be removed from the model (become 0). This reduces the number of coefficients (features) that are used by the model, making the model easier to interpret. Elastic net regressions include both L1 and L2 penalties. The weights of these penalties are tuned *via* the parameters alpha and the l1-ratio. We trained an elastic net regression model using the 54 linearly independent PC coordinates for each sample as input features (intendent variables) and chronological age as a dependent variable. For training we only used data from WT cohorts and young (day 3) mutants. Model parameters were optimized using 10-fold cross validation by Grid search over an l1-ratio from 0 to 1 in 0.01 intervals and for the alpha parameter values of [1e-5, 1e-4, 1e-3, 1e-2, 1e-1, 0.0, 1.0, 10.0, 100.0].

## Results

In this study we developed a lipid-based predictor of biological age, which we named LipidClock. The underlying data for the clock consists of the lipid profile of wildtype and mutant *C. elegans* strains, totaling 168 lipid species. The proposed LipidClock to measure the biological age of *C. elegans* consist of a data transformation *via* Principal Component Analysis followed by Elastic Net Regression. The results of this approach are described below.

### Individual Principal Components Show Correlation With Age

**FIGURE 1:**
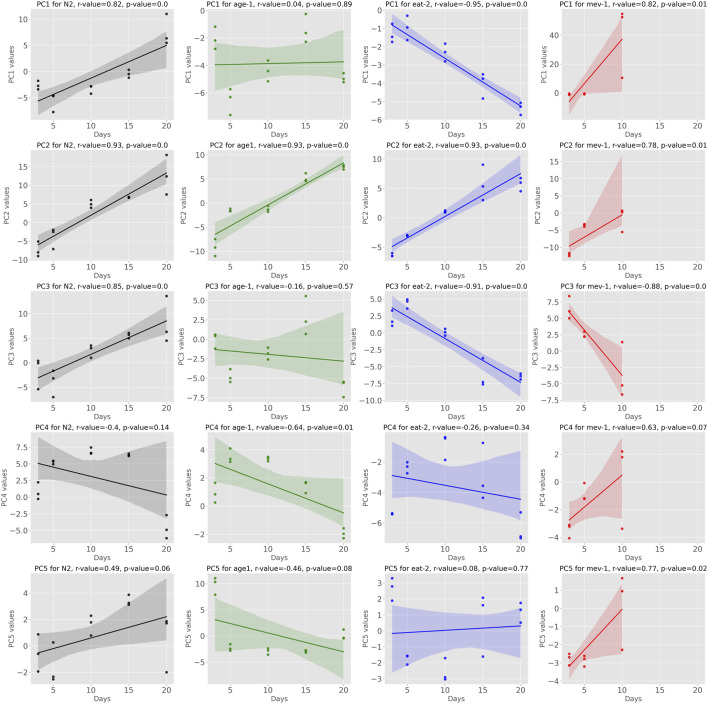
Showing the Pearson Correlation of Principal Components with age we observe that some of the PCs exhibit significant correlation with age across multiple strains. Especially PC1, PC2 and PC3 fall into this category.

By construction, coordinates along all the 54 PCs are able to reconstruct the complete dataset, reproducing 100% of the variance. However, PC1 alone captured 49% of the overall variance and, combined, the first 3, 5 and 10 PCs explained 66, 82 and 92% of the variance, respectively [Sup. Fig S1]. When analyzing data from aging cohorts, age is expected to be the major source of biological aging within each cohort (genotype, treatment condition) (Tarkhov et al., 2019; Minteer et al., 2020). Following transformation of all samples into principal component space, we therefore determined the linear correlation of each PC coordinate with age in both WT and mutant cohorts (Figure 1). We considered correlation strong when the absolute r-value was larger or equal to r = 0.7. Using this definition, we found that, among the first 5 PCs, three showed strong correlations with chronological age in WT cohorts (Figure 1). Interestingly, conservation of such patterns was variable in mutant strains, suggesting that, as expected, mutations affecting lifespan impact aging mechanisms captured by the main PCs. PC2 was unequivocally correlated to aging across all strains while PCs 1 and 3 also showed correlation with age but were less conserved across strains. Plotting PC1 against PC2 (Supplementary Figure S2) shows clusters where all the strains on day 3 are still close to each other. Furthermore, the WT at day 10 clusters with the slow aging mutants at day 15, and WT at day 15 with the mutants at day 20. However, WT at day 20 is slightly separated, but one would expect that it would cluster with *age-1* and *eat-2* at day 25 if this data were available.

Datapoints of *mev-1* on day 10 are separated from all the other data points, indicating that their trajectory has been diverging. Overall, this depicts that PC1 and PC2 encode information on aging.

**FIGURE 2:**
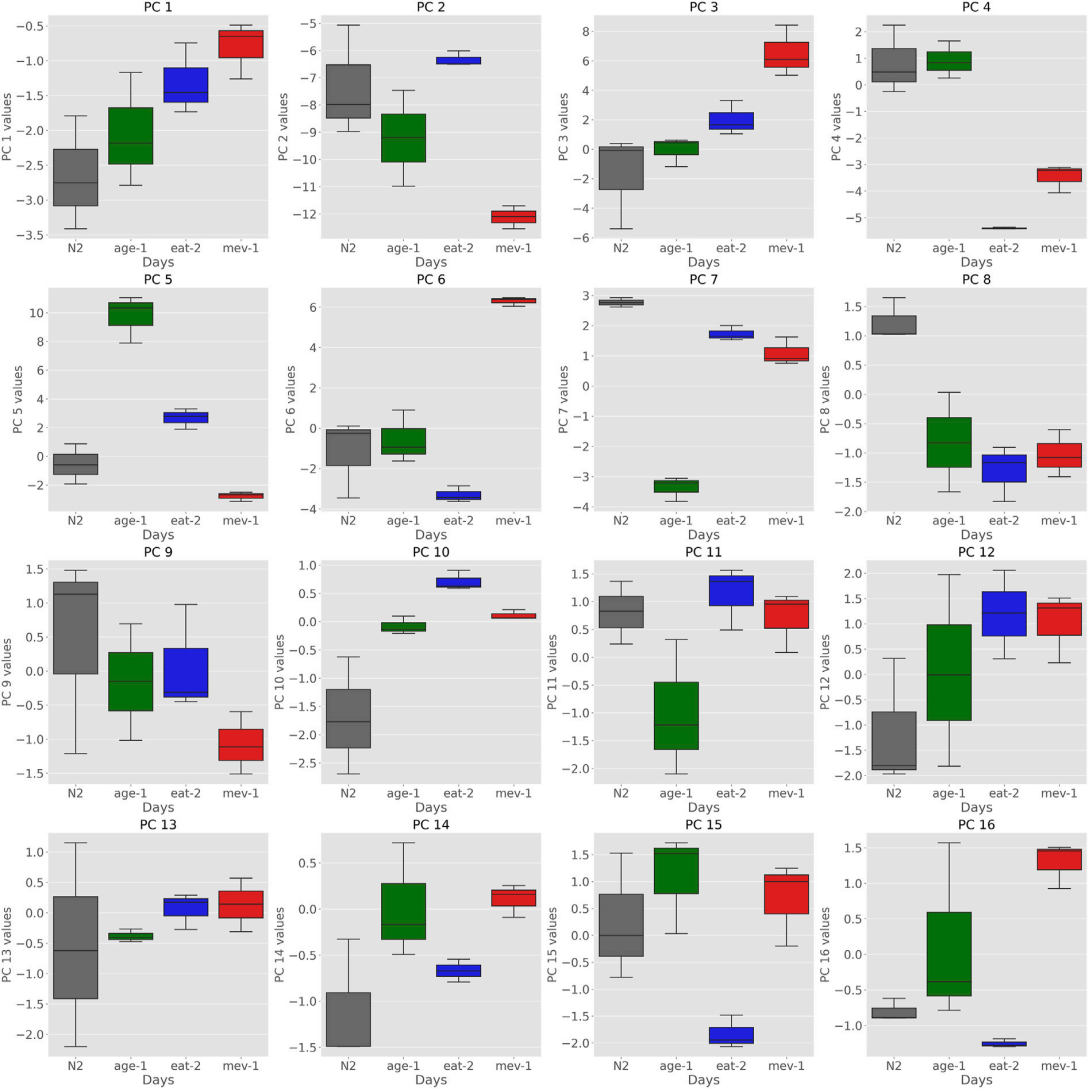
Boxplots of Principal Components of strains at day 3 show that certain PCs encode strain specific information. PCs 6, 7, and 8 encode differences in cohort related to mutant status. PC6 separates *mev-1* mutants, PC7 seperates *age-1* mutants and PC8 captured signatures for WT compared to all three mutants.

The major source of variance in our dataset when comparing samples between (rather than within) strains is mutant status. Comparing individual PC coordinates between young (day 3) WT and young mutant cohorts gives an indication which of the PCs captured initial (young) mutant status and which of these (if any) overlapped with aging PCs. Considering PCs that are not strongly correlated with aging, we found that PCs 6, 7, and 8 were clearly encoding cohort differences related to mutant status but not aging (Figure 2.). PC6 separated *mev-1* mutants, PC7 *age-1* mutants and PC8 captured signatures for all three mutants vs. WT. Neither of these PCs was strongly correlated with aging.

### The Lipid Clock

Having confirmed that several PCs capture aging changes in WT and mutant strains, we constructed an aging clock to predict cohort age based on lipid composition in terms of PCs. For this, we utilized a supervised learning method that is commonly used for the construction aging clocks, regularized linear regression (elastic-net regression).

Importantly, we carried out elastic-net regression only using wild-type samples (all ages) and samples from young (day 3) mutant cohorts. This choice meant that the model is driven to deemphasize or disregard PC coordinate directions along which mutants are already highly different from wild type when young. The resulting model therefore emphasizes directions in PC space along which WT animals exhibit substantial changes during aging but along which WT and mutants are similar when young. The model was trained to predict the chronological age of WT worms and define normal WT aging rate. Importantly, samples from old aging mutant cohorts between day 5 and day 20 were never used for training, meaning that these samples could be used as an unbiased test of the final model. We optimized L1 and L2 penalty parameters *via* Grid Search on repeated 10-fold cross validation with the training data split into 10 chunks, of which 9 were used for training and 1 for validation. This process was repeated 10 times. Grid Search found the optimal parameters to be 0.01 for alpha and 0.67 for l1-ratio.

The mean absolute error on the repeated 10- Fold cross validation was 1.45 days, meaning that within this dataset of wildtype and young (day 3) mutants, cohort age was predicted with an error of less than 2 days. The final model assigned significant weights to PC1 and PC2 with smaller contributions to higher PCs and several PCs having zero coefficients.

The Lipid Age estimator for the LipidClock is given by:

Lipid Age=8.72+0.25PC1+0.62PC2+0.05PC5+0.36PC6−0.07PC7–0.22PC9….O(PC10*) (1)

*) A full list of weights be found in Supplementary Table S3.

We then applied this lipid clock model to our day 5–20 aging mutant (*mev-1, eat-2, age-1*) cohorts to evaluate if age in mutant strains could be predicted by the model, despite the fact that no aged mutants were included in the training data.

**FIGURE 3:**
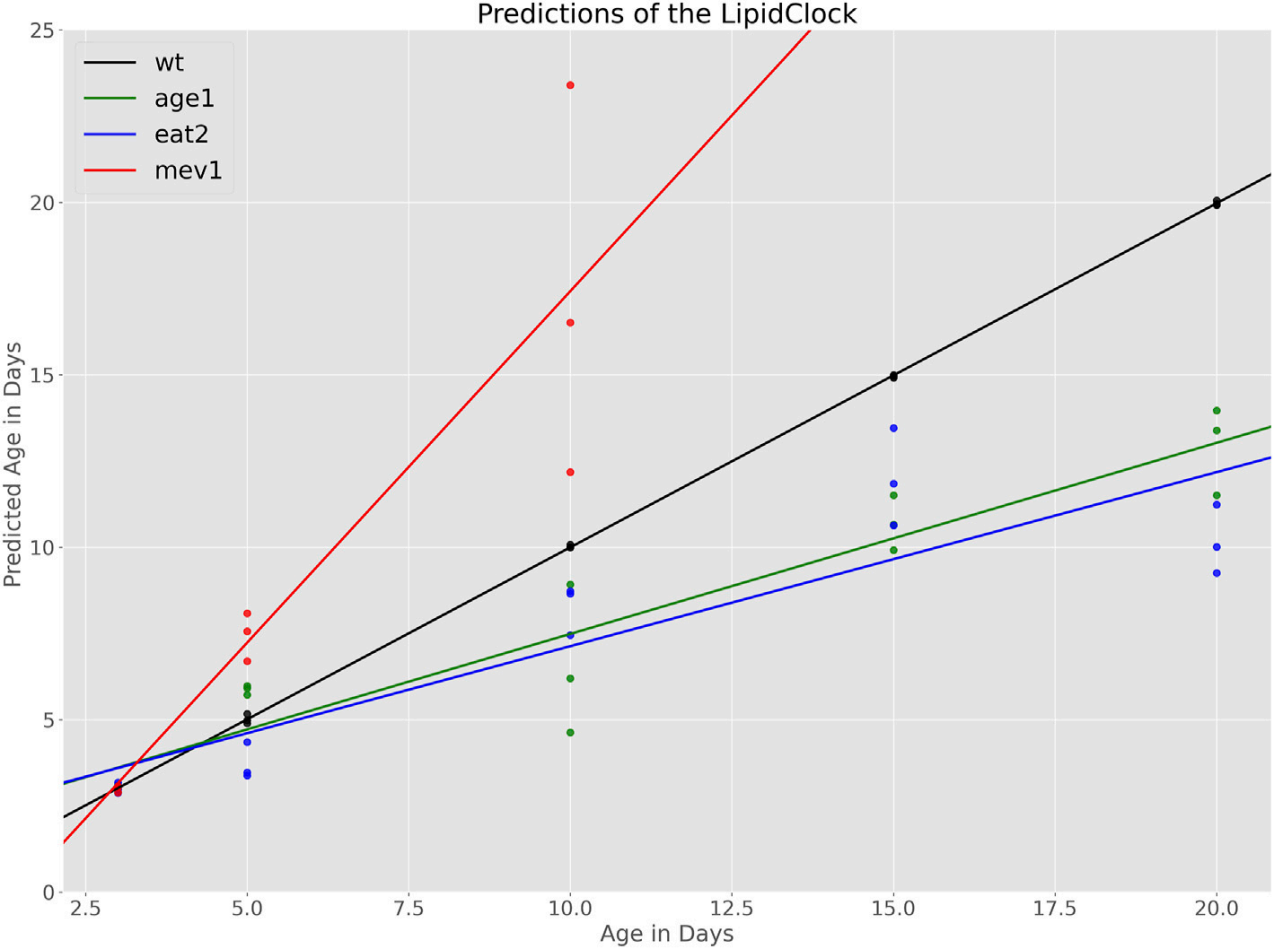
Age predictions made by the LipidClock and fitted lines indicating the aging rates. Aging rate for WT is 0.99±0.003 , for *age-1* 0.55±0.053 , for *eat-2* 0.5±0.075 , and for *mev-1* 2.04±0.003 .

Figure 3 shows the relationship between chronological age and predicted biological age for each of the mutant cohorts. Because aged WT were included in the training set, wildtype ages are predicted with high degree of accuracy, as expected. For mutant cohorts, predictions at day 3 are also highly accurate, which is also expected because young (day 3) mutants were also included in the training data. At day 5, predicted ages largely overlap for WT and the slow aging strains (*age-1* and *eat-2*). However, the model predicts substantially higher ages for *mev-1* at day 5, indicating that, based on their lipid composition, *mev-1* worms are biologically older than 5 days. From day 10 onwards, the slow aging strains *age-1* and *eat-2* followed similar trajectories to each other but are well separated from WT N2 and from the fast aging *mev-1*. Evaluating the error of the age estimator on this cohort yielded a mean absolute error of 4.13 days. This error is driven predominantly by the overestimation of ages for the *mev-1* strain. On average, the clock overestimates the age of *mev-1* by 4.9 days. In contrast, age is underestimated by 2.28 and 2.0 days for *age-1* and *eat-2*, respectively.

The rate of change (slope) of predicted biological age to actual chronological age can be interpreted as strain-specific aging rate. The aging rate for each strain was therefore determined by fitting a regression line to the relationship between chronological and predicted biological (lipid) age. As the model was trained with the chronological age of WT as dependent variable, it is not surprising that chronological age and biological agree well for WT. By construction, WT worms having an ageing slope of 1, aging 1 day per day, approximately (black line, Figure 3). Slopes below 1 indicated slower biological aging rate while slopes above one indicated accelerated aging relative to WT. Based on this definition, we find that *mev-1* age approximately twice as fast as WT (slope: 2.04±0.003 ) while *age-1* (slope: 0.55±0.053 ) and *eat-2* (slope: 0.5±0.075 ) age half as fast.

These predicted aging rates are consistent with the know biology of these strains. *Mev-1* generate excess oxidative damage and are known to be short-lived while *age-1* and *eat-2* are both long lived. However, the model does not distinguish between *age-1* and *eat-2*, even though these two strains, while both long-lived, have different lifespans.

### Simulated Survival

A defining quality of biological aging is that mortality increases exponentially with age. The age-dependent increase is mortality is described by the Gompertz–Makeham law (Eq 2.) (Gompertz, 1825):

$$M=MExt+M0e^{\ln\left(2\right)\frac{age}{MRDT}}$$ (2)

Where M is the mortality given age, MExt is the external, age-independent mortality (Makeham term), M0 is the initial mortality at the onset of adulthood, and MRDT is the mortality rate doubling time, defined as the time in days over which the risk of dying from age-dependent causes doubles.

**FIGURE 4:**
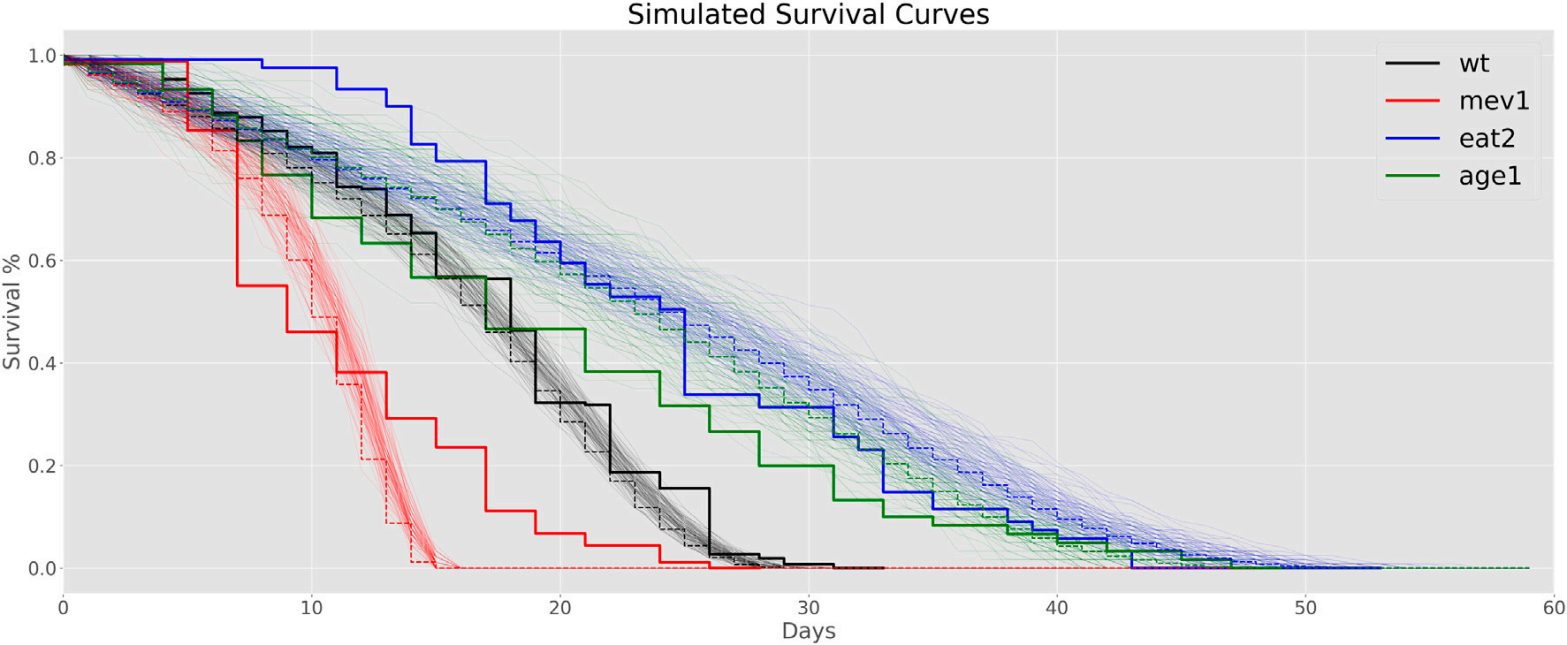
Simulated Survival Curves: Thick step curves represent data from experimental lifespan experiments. The thin lines are a total of 100 Monte Carlo simulations, with their average depicted as dotted lines.

To investigate differences in aging rate of mutants vs. WT animals further, we used the estimated aging rates to calculate mortality trajectories for each mutant strain by scaling Gompertz aging rates relative to WT (Figure 4). We then simulated lifespan data based on these scaled mortality trajectories using a simple Monte Carlo model and compared them to actual lifespan data for *mev-1* and *age-1*, collected in our laboratory under the same conditions as those used for the lipidomics cohorts (Figure 4).

We estimate MExt, M0 and the MRDT for the WT by grid search in the parameter space by calculating the root-mean square error between the average of 100 Monte Carlo simulations and the experimental data. For WT *C. elegans* MRDT at 20°C has been reported to be 3 days, hence we search for the MRDT of our data between 2.5 and 3.5 days. Furthermore, realistic values for MExt lay between 0 and 0.2, and for M0 in the interval of 0–0.01.

We determined aging rates for mutant strains relative to WT based on the biological age determined by the lipid clock and used these estimates of relative aging rates to scale the MRDT for each mutant strain. No other parameter optimization was performed, leaving MExt and M0 unchanged. In other words, we then calculated stochastic survival curves using a simple Monte Carlo approach based on the scaled strain-dependent mortality dynamics. Briefly, for each cohort and day (age), the age-specific mortality was calculated based on Eq. 2. A uniform random number between 0 and 1 was then generated for each surviving animal in the cohort. If this number was larger than the age-dependent mortality, a death event was recorded, and the number of survivors was decremented. The resulting list of death events was then analyzed using the same approach as our experimental lifespan data (Figure 4). The simulations used the same number of worms that correspond to the experimental lifespan data which were 257 *N2* worms, 60 *age-1* worms, 121 *eat-2* worms and 89 *mev-1* worms. Both the code for the LipidClock as well as for simulation of the survival curves are available on GitHub (https://github.com/max-unfried/lipid-clock).

We found that the root-mean square error between the simulated WT curves and our experimental wildtype data is low for different sets of reasonable and realistic parameter configuration. For MRDT values of 2.8–3.5 days, ExtMort between 0.01–0.02 and Mort0 in the interval of 0.002–0.005 a good fit is obtained. These parameters seem sensible as they correspond to values found in the literature.

Consistent with these estimates, WT survival in our hands is well modelled using MRDT of 3.5 days, ExtM of 1.5% per day and an initial mortality (M0) of 0.5% per day (see Figure 4.). We found that predicted lifespan curves under these conditions were also in qualitative agreement with observed survival data for *age-1* and *mev-1*, but the simulated data somewhat overestimated *eat-2* survival.

### Interpretation of PCs in Terms of Lipid Species Involved

**FIGURE 5:**
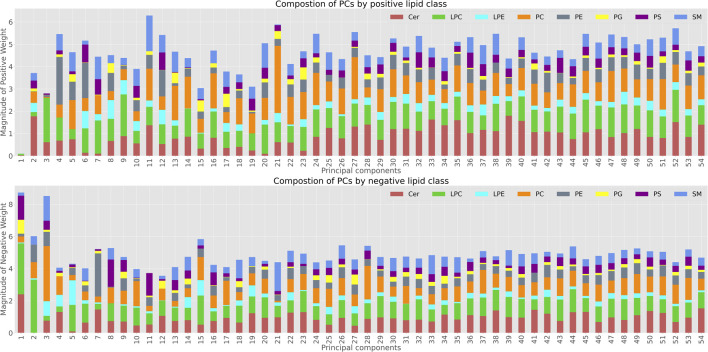
Lipid composition of Principal Components; The top panel shows the lipid species with positive weights in each principal component. The bottom graph shows the distribution of negative weighs for each of the PCs.

Transformation into PC coordinates is an orthogonal linear transformation, with each PC defining a vector in feature (lipid) space. To investigate further lipid species and pathways driving these aging changes, we grouped the individual lipid species into their main groups and analyzed contribution of individual lipid classes to each principal component (Figure 5). Each principal component is composed of a weighted sum of Cer, LPC, LPE, PC, PE, PG, PS, and SM. We separated the lipid species by positive (increase with age) and negative (decrease with age) weights.

While for many of the components there was no clear trend in terms of the lipid classes involved, by analysing the first 3 principal components and their composition we were able to determine which lipid classes were most involved in aging changes along these PCs.

PC1 was almost exclusively dominated by negative weights (decline in abundance with age), and LPC followed by Ceramides were the main lipid species involved in this decline (Figure 5).

PC2 was also mainly impacted by LPC and Ceramides, however in this case all the Ceramides contributing to PC2 had positive weights, meaning they increased with age - whereas the LPC are still weighted negatively. Moreover, aging associated LPCs such as LPC 20:4, LPC 18:2 and LPC 18:1 have high weights in PC1 and PC2 when we explore the composition of the negative weight LPC composition. PC3 appeared to capture a mixture of LPCs and PCs. Further analysis of lipid species involved, enrichment of specific species or molecular features associated with individual ageing or mutant PCs could be carried out based on these weights. However, this would be beyond the scope of this proof of principle study.

## Discussion

In this study we successfully constructed and tested a lipid clock using *C. elegans*. We used *C. elegans* because samples for ageing cohorts can be generated rapidly in this short-lived organism and because of the availability of mutant strains that differ substantially in lifespan and biological aging rate. However, the methodology described would work without major modification for lipid extracts from samples of other biological origin, including lipids extracted from blood plasma. Here, we focused on three nematode strains, two long-lived and one short lived and compare them to WT aging, demonstrating that it is in principle possible to use lipidome signatures to construct a lipid-based “clock”.

Change in an aging organism is driven by cumulative changes that are slow when compared to physiological responses, which are characterized by rapid dynamical change, such as oscillations and cycles or, following perturbation, rapid return to equilibrium. Aging is therefore expected to be a major source of variance when analyzing data describing a biological system across its lifespan. PCA is a technique to identify correlated features or “directions” in feature space along which covariance is large and this can be exploited in the construction of clocks and classifiers (Tarkhov et al., 2019; Minteer et al., 2020).

Previous works have shown that the several of the first (most important) Principal Components generally correlate with age and PC1 is considered predominantly capture aging dynamics (Tarkhov et al., 2019; Levine et al., 2020; Tarkhov et al., 2022). As expected, in our dataset, the first PCs, representing most of the variance in the data, show strong correlations with age (Figure 1.). These PCs are prime candidates for inclusion when constructing aging clocks. Indeed, 7 out of the first 10 principal components—accounting for 92% of the variance—are automatically selected by our elastic net model. However, it should be noted that not all PCs show aging patterns that are consistent between WT and mutant cohorts (Figure 1). This is expected as the mutant strains that we chose are characterised by significant perturbations in food intake (*eat-2*), aging rate and stress response (*age-1*) and mitochondrial metabolism/oxidative damage (*mev-1*). All have lifespans substantially different from WT.

Despite 1) these differences and 2) the comparatively small dataset, our lipid clock was able to identify both acceleration and slowing of biological aging in these mutant strains. The model not only correctly identified strains that age faster or slower in terms of their biological (lipid) age compared WT, but these data can be used to scale survival curves and predict lifespans with relatively high accuracy (Figure 4.). It is noteworthy that in constructing the clock, we never made use of any of the aged mutant samples. At no point did we encode remaining lifespan or strain-specific morality as training variable. Even though the model therefore had never “seen” data on biological aging for any of the mutant strains, it correctly identified fast aging *mev-1* as biologically older than their chronological age and slow-aging *age-1*/*eat-2* as biologically substantially younger. This indicates that aspects of aging have signatures in lipid space that are universal or at least preserved between WT and diverse mutant strains.

In this study, we did not focus on the specific changes in lipid species, but rather on the global changes in lipid space that can correlate with lifespan. Further analysis of these changes to the lipidome may provide insights into signalling membrane composition and metabolic changes involved in aging with specific lipids involved in specific physiological process (de Diego et al., 2019).

Analysing the principal components and their weighted composition of lipid species, we can give indications on the interpretations of these PCs. If a lipid has a negative weight in a PC, and the PC is positively correlated with age, the lipid will decrease with age. If the PC is negatively correlated with age, and the lipid has negative weights, the lipid will increase. For positive weights the reverse is true. Analysing the composition of the individual PCs, we find that for the 1st PC almost all lipid species have a negative weight and PC1 is the only PC for which this is the case. PC1 being positively correlated with age, having a Pearson correlation coefficient of r = 0.82 for the wildtype, means that PC1 captures lipid species that are correlated with each other and that decrease with age in the wildtype.

PC2 is both positively correlated with age for all the strains and has a significant weight in the elastic net regression. Analysis of the lipid species involved in PC2 showed an age-dependent increase in certain Ceramide species and a decrease in LPCs. This is consistent with recent studies that describe an increase of Ceramides (Sacket et al., 2009; Khayrullin et al., 2019) and a decrease of LPCs with age (Gonzalez-Freire et al., 2019). Furthermore, LPC 20:4, LPC 18:2 and LPC 18:1 are associated with aging phenotypes such as LPC 18:2 being a strong predictor of accelerated decline in gait speed (Gonzalez-Freire et al., 2019), low LPC 20:4 is associated with decreased mitochondrial oxidative capacity (Semba et al., 2019), and LPC 18:1 being a biomarker for human longevity with higher concentrations in the plasma of centenarians (Montoliu et al., 2014). In our case, LPC 18:1 and LPC 20:4, have strong negative weight in the first Principal Component, whereas LPC 18:2 has the second largest negative weight in PC2, while LPC 18:1 is still weighted heavily.

Consistent with other recent developments in aging clocks, we find that dimensionality reduction by PCA prior to regression functions well in constructing aging clocks (Minteer et al., 2020). One drawback of PCA and linear regression methods is that they can only capture linear dependencies. However, signatures of non-linear dynamics may be accounted for by further refinement, for example using Mutual Information scaled PCA, kernel methods or neural networks to extract more representative features. Such approaches would however require substantially larger datasets if available, but may further enhance the predictive power.

Our findings suggest that lipid clocks may be a promising addition to the emerging field of -omics-derived aging clocks. Lipids are a diverse class of molecules that play a pivotal role in many physiological processes. A limitation of this study is that it is currently based on whole body lipid extracts while lipids clocks suitable for human studies would have to be based on lipid signatures available based on less invasive samples, in particular lipid extracts from body fluids such as plasma or urine. However, lipids derived from human blood serum are affected by physiological and pathophysiological process throughout the body. There is an abundant existing literature regarding specific blood lipids and lipid signatures and their link with aging, disease risk and disease processes (Ridker et al., 2005; Shah et al., 2008; Arsenault et al., 2011; Gonzalez-Covarrubias et al., 2013; de Diego et al., 2019; Lim et al., 2020). Lipid clocks may therefore be an attractive option for systems-level readouts of age-dependent metabolic and disease status that can be constructed using a class of molecules accessible in body fluids.

## Conclusion

In this study we developed to our knowledge the first lipid based predictor of biological age and named it “LipidClock”. This is a proof-of-principle study demonstrating that lipid composition can be used to infer biological age in a similar way as can DNA methylation or transcriptional changes. The LipidClock consists of the application of Principal Component Analysis to transform (rotate) coordinates in feature space, followed by Elastic Net Regression. Applied to the nematode *C. elegans* and trained on ageing changes in the lipid composition of WT animals, yielding a Mean Absolute Error of 1.45 days in WT animals. Furthermore, this approach successfully infers biological age of the two long-lived mutant strains, although the model over-estimates ageing in the fast ageing *mev-1* strain.

Using these predicted aging rates for mutant strains to simulate survival curves, we find that the predicted survival curves qualitatively align with empirical survival for the same strains. This analysis shows that information on the aging rate of *C. elegans* is encoded in the lipidome and that key aspects of these lipid-encoded ageing patterns are conserved between WT animals and mutant strains, even though these mutants experience substantially different lifespans. Using the same approach, LipidClocks could be developed for other organisms, including other invertebrate models, mammals or (based e.g., on blood lipids) humans. In addition to their potential use as age-classifiers, such clocks might provide insight in lipid related mechanisms of ageing. While we did not carry out in-depth analysis in terms of specific lipid classes, lipid species or mechanisms, such information is encoded in the rotation matrix of the PC analysis and in the weights in the clock classifier. These data could be used as basis for “lipid-set enrichment” or pathway analysis in a similar fashion to gene-set and pathway enrichment analysis in the context of transcriptional data, although the relevant tools are not yet as well developed for lipid analysis. Fundamentally, as other have pointed out, it is likely that similar “omics clock” approaches can be fruitfully applied to many different types of high-dimensional ageing data.

## Data Availability

The original contributions presented in the study are included in the article/Supplementary Material, further inquiries can be directed to the corresponding author. In addition the data is uploaded at https://github.com/max-unfried/lipid-clock.
