# Supplementary material for: LipidClock: A Lipid-Based Predictor of Biological Age
Source: Front Aging. 2022 May 27;3:828239. doi: 10.3389/fragi.2022.828239 (PMC9261347; doi:10.3389/fragi.2022.828239)
Supplement: Supplementary file 1 [file DataSheet1.pdf]

Supplementary Material

Table S1: For the following days and lipid species we did not collect lipidomic data and hence interpolated the missing values with the average value for each respective species.

Day	Strains	Interpolated Lipid Species
3	*N2*<br>*age-1*<br>*eat-2*<br>*mev-1*	PS 34:1, PS 34:2, PS 36:4, PS 36:5, PS 34:2.1, PS 36:1, PS 36:2, PS 40:7, PS 38:0, PS 38:4, PS 38:5, PS 40:12, PS 40:6
10	*mev-1*	SM 31:1, SM 34:1, SM 35:0, SM 36:3, SM 36:2, SM 36:1, SM 36:0, SM 38:4, SM 38:3, SM 38:2, SM 38:1, SM 38:0, SM 39:0, SM 40:1, SM 40:0, SM 41:2, SM 41:1, SM 42:2, SM 42:1, SM 42:0<br><br>PC 28:1, PC 29:0, PC 29:1, PC 30:0, PC 30:1, PC 31:1, PC 32:0, PC 32:1, PC 32:2, PC 32:3, PC 33:1, PC 34:1, PC 34:2<br>PC 34:3, PC 34:4, PC 34:5, PC 35:1, PC 36:0, PC 36:1, PC 36:2, PC 36:3, PC 36:4, PC 36:5, PC 36:6, PC 38:0, PC 38:1, PC 38:2, PC 38:3, PC 38:4, PC 38:5, PC 38:6, PC 38:7, PC 40:0, PC 40:1, PC 40:4, PC 40:5, PC 40:6, PC 40:7, PC 40:8, PC 44:0, PC 44:2, PE 30:1, PE 32:0, PE 34:1, PE 34:2, PE 34:3, PE 34:4, PE 34:5, PE 35:1, PE 36:0, PE 36:1, PE 36:2, PE 36:3, PE 36:4, PE 36:5, PE 38:3, PE 38:4, PE 38:5, PE 38:6, PE 40:5
10	*eat-2*	PE 30:1, PE 32:0, PE 34:1, PE 34:2, PE 34:3, PE 34:4, PE 34:5, PE 35:1, PE 36:0, PE 36:1, PE 36:2, PE 36:3, PE 36:4, PE 36:5, PE 38:3, PE 38:4, PE 38:5, PE 38:6, PE 40:5

Table S2: Samples collected
For each strain, samples were collected in triplicate at each timepoint. For WT N2 and both of the long-lived *age-1* and *eat-2* strains, triplicate (3) samples were collected on day 3, 5, 10, 15 and 20 of life. For the short-lived *mev-1* strain, samples were only collected up to (including) day 10 as animals of this strain start to die off after this age.

	**Age:**	3 days	5 days	10 days	15 days	20 days		
**Strain**								**Samples**
N2 WT		3	3	3	3	3		15
age-1		3	3	3	3	3		15
eat-2		3	3	3	3	3		15
mev-1		3	3	3	0	0		9
**Samples**:		12	12	12	9	9		**Total:** 54

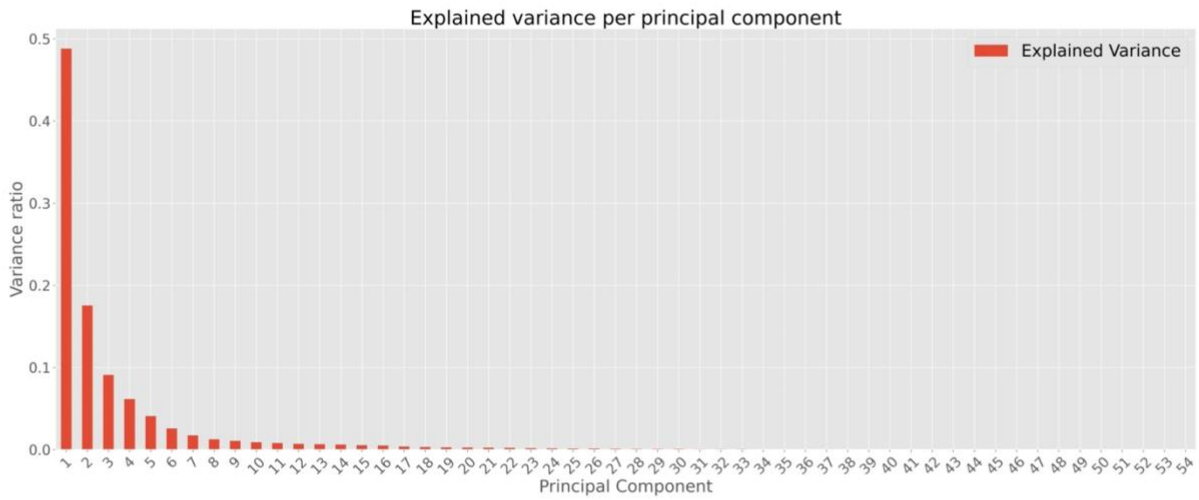

Figure S1: Explained Variance by each principal component

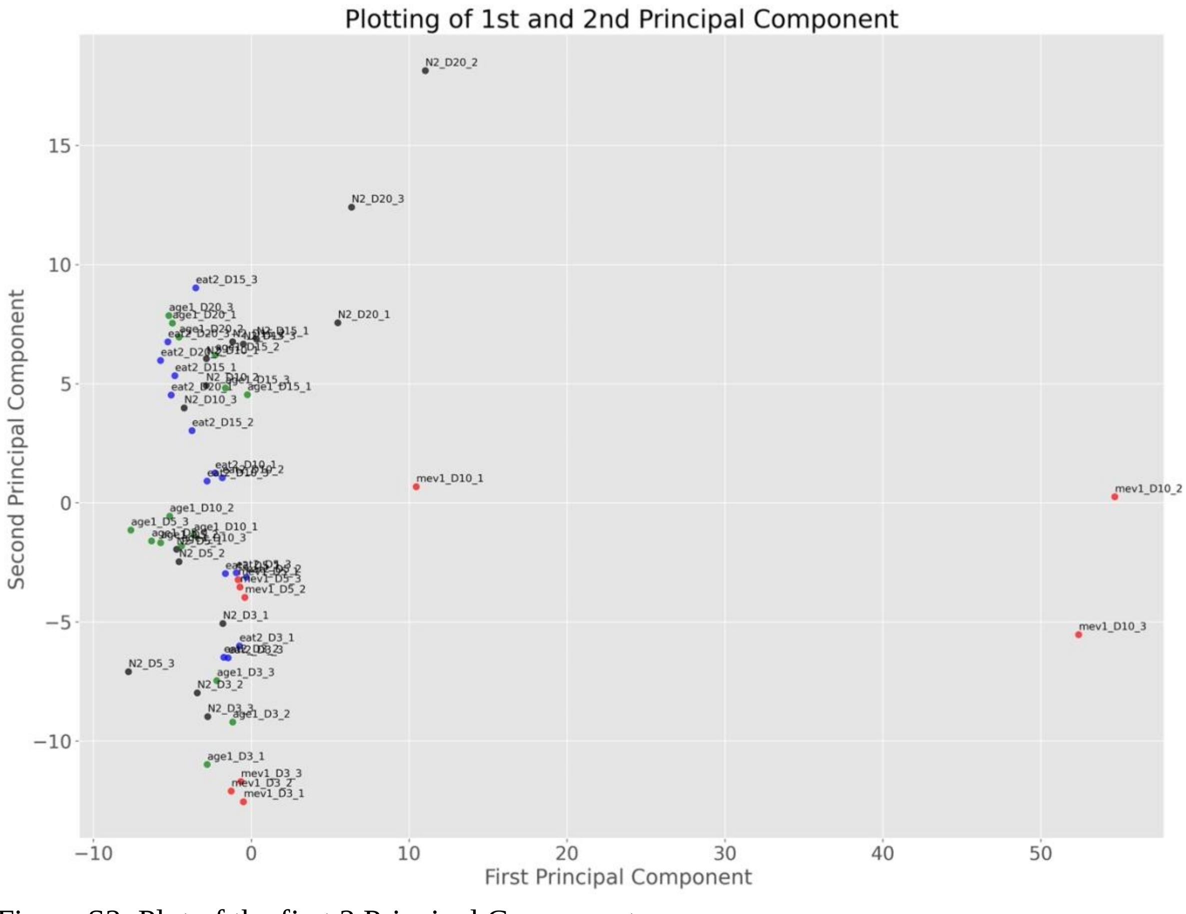

Figure S2: Plot of the first 2 Principal Components

Table S3: Weight assigned for each principal component by the elastic net model:

Principal Component	Weight of the Principial Component in the model
1	0.24493956
2	0.61887164
5	0.05166389
6	0.36068704
7	-0.07221047
9	-0.22351026
10	0.16122654
12	-0.34113561
13	0.06172371
15	0.44233765
18	-0.09540104
19	0.24626368
20	0.86748846
21	0.59345682
22	-0.6973369
23	-0.76140243
24	-0.5191236
25	-0.0309329
27	-0.10155841
33	-0.29564258
39	-0.1586736
42	-0.46037303
46	-0.13997863
52	0.12513804
Intercept	8.727458050992002
